# Damage Detection Performance of the Electromechanical Impedance (EMI) Technique with Various Attachment Methods on Glass Fibre Composite Plates

**DOI:** 10.3390/s19051000

**Published:** 2019-02-26

**Authors:** Rudy Tawie, Hee Beom Park, Jongdae Baek, Wongi S. Na

**Affiliations:** 1 Faculty of Civil Engineering, Centre for Engineering Studies, Universiti Teknologi MARA, Kota Samarahan 94300, Malaysia; rudy@sarawak.uitm.edu.my; 2 Department of Infrastructure Safety Research, Korea Institute of Civil Engineering & Building Technology, Gyeonggi-Do 10223, Korea; heebeompark@kict.re.kr (H.B.P.); jdbaek@kict.re.kr (J.B.)

**Keywords:** composite materials, non-destructive testing, electromechanical impedance technique, delamination, crack damage

## Abstract

Composite materials such as glass and carbon fibre composites have become popular and the preferred choice in various applications due to their many advantages such as corrosion resistance, design flexibility, high strength and light weight. Combining materials with different mechanical properties make composites more difficult to evaluate where the damage mechanisms for composites are more complex than traditional materials such as steel. A relatively new non-destructive testing (NDT) method known as the electromechanical impedance (EMI) technique has been studied by various researchers, but the damage detection performance of the method on composite structures still requires more investigations before it can be accepted for field application, especially in aerospace industry due to the high standard of safety. In this paper, the detection capabilities and performance of the EMI technique subjected to different PZT attachment methods have been investigated. To this end, glass fibre composite plates with various attachment methods for the sensor have been prepared and detection of common defects such as delamination and crack with the EMI technique under study has been performed. The performance of each attachment method for identifying different damage types has been analysed and finite element analysis (FEA) was carried out for verification of the experimental results.

## 1. Introduction

Composite materials such as glass and carbon fibre composites are a category of non-metallic materials that is growing in popularity. Although more expensive than common metals such as steel and aluminum, new applications for composites are increasing every day in various fields (including aerospace, automotive and civil structures) due to their advantages such as corrosion resistance, design flexibility, high strength and light weight. In terms of quality control, composites are more difficult to evaluate than traditional materials (such as steel) as materials with different mechanical properties are combined to create a composite material. As with other traditional materials, defects can occur during the manufacturing process or the service life of the materials. Damage mechanisms for composites are more complex due to different damage distribution and development mechanisms that can occur [1]. Defects such as delamination and cracks may occur due to impact or fatigue stresses and detecting these damages at an early stage is crucial to ensure safety, especially in the aerospace industry. Various non-destructive testing (NDT) techniques have been developed for testing composite structures, which can be generally categorized as contact and non-contact methods. Contact methods such as traditional ultrasonic testing, eddy current testing, and electromagnetic testing [2,3,4] require a sensor to be physically attached to the host structure for acquiring reliable data, while non-contact methods that use technologies such as infrared, radiography, thermography and holography [5,6,7,8] have an advantage in situations where physical contact between the sensor and tested structure is not possible.

In this study, a relatively new and low cost NDT technique is used for damage detection on composite plates made of glass fibre and epoxy. The method, which will be explained in the next section, is widely known as the electromechanical impedance (EMI) technique [9,10,11,12,13,14] and there are a variety of ways for performing the EMI measurements. In general, a small sized piezoelectric transducer (lead zircornate titanate – PZT) is attached to a host structure for damage identification. However, the possibility of successful damage detection for concrete or composite structures can be very low due to the absence of a resonance frequency range. In addition, the brittle nature of PZT makes it harder for one to attach it to curved structures such as pipelines. To overcome these problems, various PZT attachment methods have been investigated to date. A few of the latest research results on this these methods can be found in [15,16]. Thus, in this research, the damage detection performance of these different PZT attachment methods is presented for various damage scenarios. Furthermore, one of the PZT attachment methods has been investigated using finite element analysis to achieve a deeper understanding of the approach.

## 2. Electromechanical Impedance (EMI) Technique

Equation (1) shows that the electrical admittance Y(ω) is a combined function of the mechanical impedance of the host structure $Z_{s}\left(\omega \right)$ and the PZT transducer $Z_{a}\left(\omega \right)$, respectively. This one-dimensional equation was first introduced by Liang et al. [17] to show that any change in the host structure can be monitored by measuring Y(ω). Other variables in the equation I, V, ω, a, $\varepsilon_{33}^{T}$, δ, $d_{3x}$, ${\bar{Y}}_{xx}^{E}$ are the PZT output current, PZT input voltage, input frequency, geometric constant, dielectric constant, loss tangent, piezoelectric constant and Young’s modulus, respectively:

(1) $$Y\left(\omega \right)=i\omega a\left(\varepsilon_{33}^{T}\left(1-i\delta \right)-\frac{Z_{s}\left(\omega \right)}{Z_{s}\left(\omega \right)+Z_{a}\left(\omega \right)}d_{3x}^{2}{\bar{Y}}_{xx}^{E}\right)$$

**Figure 1:**
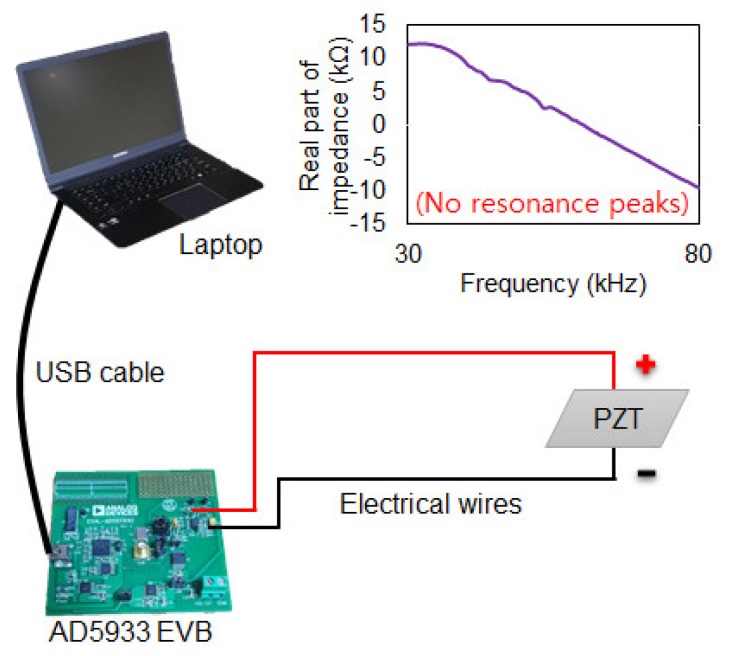
EMI measurement performed using an AD5933 evaluation board.

The EMI technique can be performed using commercial impedance analyzers such as the 4294A and E4990A which are generally used to perform the technique. Another method of conducting the EMI technique is to connect a function generator with an oscilloscope whereby more details can be found in [18]. For this study, the EMI technique was performed using an AD5933 evaluation board manufactured from Analog Devices Co. (Norwood, MA, USA) The evaluation board can measure up to 100 kHz with 512 maximum data points. Figure 1 shows that the device can be connected to a laptop and powered via the USB cable where the EMI measurement can be performed by using the software provided by Analog Devices. In a conventional EMI measurement setup as shown in Figure 1, a PZT (model 5A4E, purchased from Piezo.com, Woburn, MA, USA), is used as a sensor and attached to a target structure with the positive and negative wires from the AD5933 board connected to it. Impedance signatures are acquired before and after damage is induced in the target structure and the damage intensity can be quantified by using statistical metrics such as root mean square deviation (RMSD), mean absolute percentage deviation (MAPD) and correlation coefficient deviation (CCD). The impedance signature shown in Figure 1 is a sample result of EMI measurement. Generally, for the EMI technique to be successful in detecting damage, the impedance signature must have multiple peaks (resonance). As can be seen in Figure 1, no clear peaks exist throughout the frequency range of the impedance signature, hence such a signature will most likely fail when used to detect any damage in the target structure. This illustrates a common problem of conducting EMI technique on composite structures or on concrete as aforementioned in the previous section.

## 3. Various PZT Attachment Methods

**Figure 2:**
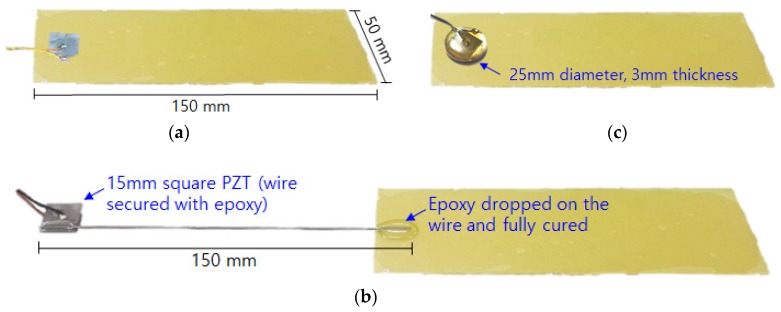
Various PZT attachment methods. (**a**) Conventional PZT attachment; (**b**) Steel wire-PZT attachment; (**c**) Metal disc-PZT attachment.

**Figure 3:**
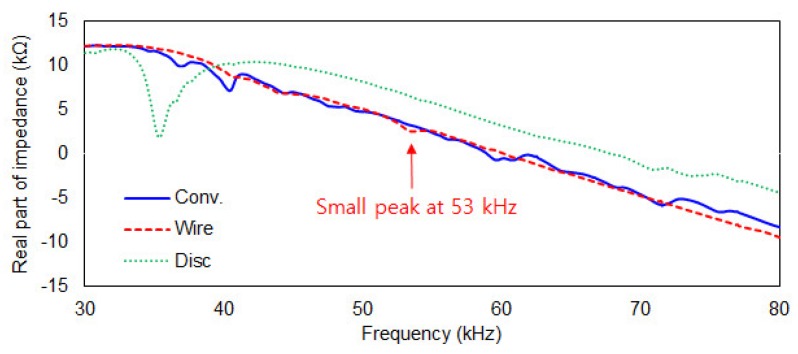
Reference impedance signatures for the three different PZT attachment methods.

In this study, epoxy fibre glass laminated sheet (with tensile strength over 300 MPa) of 150 mm × 50 mm × 0.15 mm purchased from the domestic company Artryx (www.artryx.com; Gyeonggi-Do, South Korea) was used as the target structure. EMI measurement was performed with a frequency range from 30 to 80 kHz using the low cost AD5933 board in 100 Hz steps. Figure 2 shows the different PZT attachment methods, while samples of the acquired impedance signatures for the various PZT attachment methods prior to damages being induced in the composite plates are shown in Figure 3. The three attachment methods are explained in details in the following subsections.

### 3.1. Conventional PZT Attachment

As explained previously, the presence of multiple peaks throughout the frequency range of measured impedance signature is important for the EMI technique to be effective in detecting any damage. Hence, a thinner composite plate was used in this study to allow presence of multiple peaks using the conventional method of PZT attachment as shown in Figure 2a. The 15 mm × 15 mm × 0.508 mm sized PZT was attached to the near end of the composite plate using a commercial epoxy (Quick-set Epoxy, Loctite, Düsseldorf, Germany) and left for 48 h at room temperature to ensure full curing of the adhesive. The damage detection performance of conventional PZT attachment method on the composite plate would be later compared to other methods in Figure 2b,c. Observing the blue line in Figure 3, multiple peaks with small amplitudes can be seen exist throughout the measured frequency range. Compared to Figure 1 where the impedance signature is peakless, a thinner plate can result in better performance when conducting the EMI technique due to existence of multiple peaks. This is a vital factor when analysing the signature for damage identification as these peaks will change subjected to any change in the structure property.

### 3.2. Steel Wire-PZT Attachment

As shown in Figure 2b, a PZT patch was attached to a steel wire (thickness 0.5 mm) where the other end of the wire was attached onto the composite plate using the epoxy adhesive. The idea of using steel wire for EMI technique was first proposed in [19] where the purpose was originally to overcome the problem of conducting the EMI measurement on structures with complex shapes and to allow the EMI technique to be performed on any type of surfaces as only the tip of the steel wire needs to be attached onto the target structure. For the second impedance signature (red line) labelled “Wire”, the signature is virtually peak-free with a very small peak at around 53 kHz can be seen (Figure 3). Generally, when performing the EMI measurement, such an impedance signature should be avoided. For this study, it is interesting to find out how this affects performance of the steel wire-PZT attachment method. Hence, the EMI technique for this case was still carried out regardless of the shape of the acquired impedance signature to evaluate its damage detection performance.

### 3.3. Metal Disc-PZT Attachment

Figure 2c shows another way of performing the EMI measurement using a PZT patch that was attached onto a metal disc which was then secured onto the target structure. For this approach, the size of the metal disc used for this study was 25 mm in diameter with 3 mm thickness. This attachment method was also supposed to overcome the problem of peakless impedance signatures which can occur when conducting EMI technique for composite structures. It is possible to create resonance in the known frequency range and details of this can be found in [20]. In Figure 3 for the third impedance signature (green line) labelled “Disc”, one large peak exists in the frequency range of around 35 kHz and also a small resonance can be seen above 70 kHz. However, the large peak could be the resonance of the metal disc. Thus, the effectiveness of this attachment method for identifying damages was investigated and compared with the other two attachment methods in this study.

## 4. Experimental Setup for Damage Detection Performance

**Figure 4:**
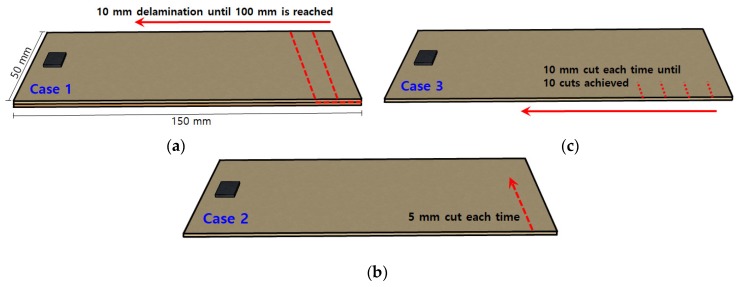
(**a**) Case 1—Delamination defect between two composite plates; (**b**) Case 2—Increasing crack damage at fixed location; (**c**) Case 3—Progressive crack damage towards the PZT patch.

In this section, the different damage types and how the EMI measurements were carried out are explained. Figure 4 shows the summary of the damage types that were introduced for this study. For all the experiments, the frequency range was measured from 30 kHz to 80 kHz in 100 Hz step where the intact case impedance signature was used as the reference signature with all other signatures (damaged) were compared to this reference. After acquiring all the experimental data, the impedance signatures were analysed using three different statistical metrics mentioned earlier. The full equations for these statistical metrics can be found in the later section.

### 4.1. Delamination Defect between Two Composite Plates

**Figure 5:**
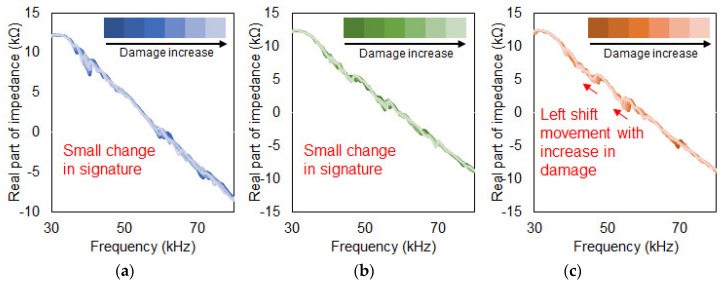
Delamination damage. (**a**) Conventional PZT attachment; (**b**) Steel wire PZT attachment; (**c**) Metal disc PZT attachment.

The first damage type (Case 1) shown in Figure 4a is known as delamination defect where two composite plates (0.15 mm thickness each) that were adhered together with epoxy was artificially separated or delaminated using a chisel. 10 mm in length was delaminated each time (50 mm × 10 mm, 500 mm^2^ each time) with the impedance signatures being measured until 100 mm of delamination was reached (50 mm × 100 mm, 5000 mm^2^ delaminated in total). This resulted in 11 impedance signatures including the reference signature that was acquired before the damage was induced or at intact state. Then, this test was repeated for the other two different PZT attachment methods (Section 3.2 and Section 3.3) where the impedance signatures can be seen in Figure 5.

### 4.2. Increasing Crack Damage at a Fixed Location

**Figure 6:**
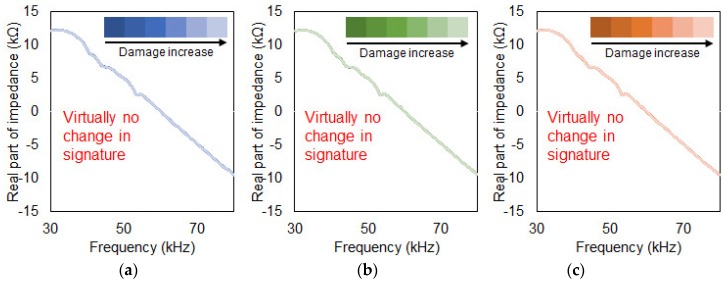
Increasing damage at a fixed location. (**a**) Conventional PZT attachment; (**b**) Steel wire PZT attachment; (**c**) Metal disc PZT attachment.

In the second damage type (Case 2) as shown in Figure 4b, a 5 mm crack was artificially created using a cutter until a depth of 50 mm was reached. Impedance signatures were measured at every 5 mm cut until the end part of the composite plate was completely removed. The reason for this test was to evaluate the damage detection ability with increasing damage occurring at a fixed location. A total of 11 impedance signatures were acquired including the reference signature. The same test was repeated for the other two different PZT attachment methods where the impedance signatures can be seen in Figure 6.

### 4.3. Progressive Crack Damage towards the PZT Patch

**Figure 7:**
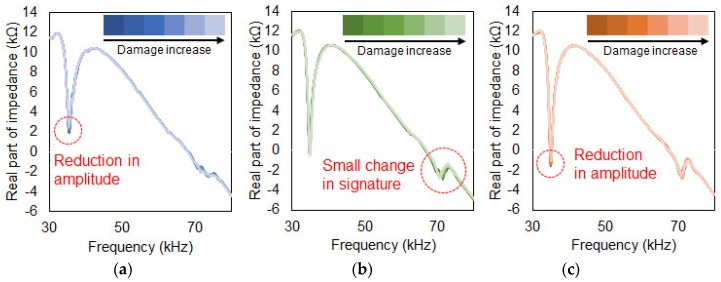
Progressive crack damage towards the PZT. (**a**) Conventional PZT attachment; (**b**) Steel wire PZT attachment; (**c**) Metal disc PZT attachment.

The last experiment (Case 3) aimed to create an example of progressive damage in the direction towards the PZT patch as shown in Figure 4c. A 10 mm crack was first made with the impedance signatures being measured after each additional cut until 10 cuts were made towards the PZT transducer, totaling 11 impedance signatures including the reference signature. Then, the same test was conducted for the other two different PZT attachment methods where the impedance signatures are shown in Figure 7.

## 5. Discussion and Results

Figure 5, Figure 6 and Figure 7 show the acquired impedance signatures for the experiments conducted in the previous section. Figure 5 shows the results for the three different damage types for the tests with conventional PZT attachment methods (Section 3.1). Observing the figures, the changes in the impedance signatures can be clearly identified. The peaks in the signatures seem to change randomly when subjected to damage without any clear pattern. For Figure 5c two of the peaks in the figure shift in the left direction as damage intensity increases. Figure 6 shows the results for the experiment for the PZT attached to the composite plate using a steel wire (Section 3.2). By observation for all three figures, virtually no change in the impedance signatures can be found subjected to damage regardless of the different damage types. Since the PZT is attached using a steel wire, one can assume that the sensitivity of this approach is very low when subjected to damage, resulting in virtually no change in the impedance signatures. More on this will be discussed in the later section with the results obtained using the statistical equations. Figure 7 shows the outcome from the experiment using a metal disc sandwiched between the PZT patch and the host structure (Section 3.3). One large peak exist at around 35 kHz which is the resonance of the metal disc where the amplitude reduces subjected to damage for Figure 7a,c.

For evaluating the damage detection performance, the variations between the reference and corresponding signatures (after damage) were quantified using three common statistical metrics in equations below. In the equations, $Re\left(Z_{i}\right)$ and $Re\left(Z_{i}^{o}\right)$ represents real part of the reference and the corresponding impedance signatures, respectively, N is the number of impedance signatures and symbols $\bar{Z}$ and $\sigma_{Z}$ signifies mean values and standard deviations, respectively:

(2) $$\mathrm{RMSD}=\sqrt{\sum_{N}{\left[Re\left(Z_{i}\right)-Re\left(Z_{i}^{o}\right)\right]}^{2}/\sum_{N}{\left[Re\left(Z_{i}^{o}\right)\right]}^{2}}$$

(3) $$\mathrm{MAPD}=\sum_{N}\left|\left[Re\left(Z_{i}\right)-Re\left(Z_{i}^{o}\right)\right]/Re\left(Z_{i}^{o}\right)\right|$$

(4) $$\mathrm{CCD}=1-\mathrm{CC},\;\mathrm{where}\;\mathrm{CC}=\frac{1}{\sigma_{Z}\sigma_{Z^{o}}}\sum_{N}\left[Re\left(Z_{i}\right)-Re\left(\bar{Z}\right)\right].\left[Re\left(Z_{i}^{o}\right)-Re{\bar{Z}}^{o})\right]$$

The calculated RMSD, MAPD and CCD values for the three PZT attachment methods are given in Table 1, Table 2 and Table 3. The evaluation of the damage detection performance of each method based on the three metrics above is discussed in the sub-sections below.

### 5.1. Damage Detection Performance of Conventional PZT Attachment Method

**Figure 8:**
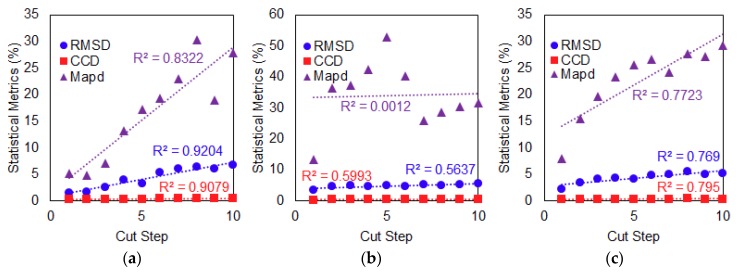
Performance evaluation of conventional PZT attachment method subject to damage (**a**) Case 1; (**b**) Case 2; (**c**) Case 3.

Figure 8 shows the plotted best-fit line of the calculated RMSD, MAPD and CCD values versus the number of cut steps and the R^2^ coefficient used for evaluating performance of the conventional PZT attachment method. For the first damage type (Case 1), a positive correlation can be seen in Figure 8a, where the RMSD, MAPD and CCD values increase as the delamination defect is increased. Based on the plotted best-fit line, all values of the three metrics show a strong linear relationship with increasing delamination defect where their calculated R^2^ coefficient is more than 0.8. In Figure 8b for the second damage type with increasing crack damage at a fixed location (Case 2), the calculated R^2^ coefficient for RMSD and CCD values is near to 0.6 which shows that the trend is positive but can be considered only fair or moderate in terms of degree of correlation with the increasing crack damage. For the MAPD metric, it can be said that there is no relationship between the MAPD values and increasing crack damage since its R^2^ coefficient is near to zero (0.0012). For the third damage type (Case 3), which is progressive crack damage towards the PZT patch, it can be noted that the final values of the three metrics look similar to values in Case 1. All three metric values can be seen to have a strong linear relationship when compared with increasing progressive crack damage as shown in Figure 8c where their calculated R^2^ coefficient is near to 0.8. Overall, the performance of the conventional PZT attachment method was quite good especially for Case 1 and Case 3 damage types when the damage was quantified using the three different statistical metrics. It is important to state here that the use of a thinner plate had influenced the performance of the conventional attachment method where the existence of multiple peaks in the frequency range of the impedance signatures had resulted in good performance of the conventional PZT attachment method.

### 5.2. Damage Detection Performance of the Steel Wire-PZT Attachment Method

**Figure 9:**
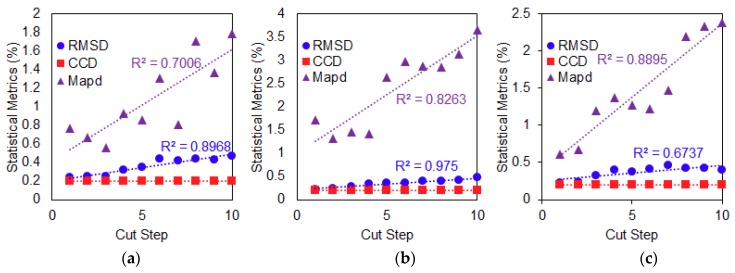
Performance evaluation of steel wire PZT attachment method subject to damage (**a**) Case 1; (**b**) Case 2; (**c**) Case 3

Performance of the steel wire-PZT attachment method with regard to the three different damage types can be seen in Figure 9. For Case 1 as shown in Figure 9a, the RMSD values have the strongest correlation with increasing delamination defect with R^2^ = 0.8968 followed by MAPD with R^2^ = 0.7006. For Case 2 with increasing crack damage at fixed location (Figure 9b), the RMSD values with R^2^ = 0.975 again show better correlation than MAPD with R^2^ = 0.8263. Only for Case 3 with progressive crack damage towards the PZT patch (Figure 9c) that the MAPD values with R^2^ = 0.8895 show better correlation than RMSD with R^2^ = 0.6737. For CCD values versus delamination defect, its R^2^ coefficient is virtually zero in all three damage cases where the calculated CCD values are the same for all the cut steps as shown in Table 2 and therefore, it can be said that no relationship exists between the two variables. It is worth noted that the performance of steel wire-PZT attachment method in detecting increasing crack damage at a fixed location with RMSD and MAPD metrics is much better than the conventional PZT attachment method using the same metrics, with the values of their R^2^ coefficient are greater than the latter. The CCD metric, however, is not recommended when used to quantify damage in composite plates for the steel wire-PZT attachment method because the results show no relationship exist between CCD values and any of the three damage types.

### 5.3. Damage Detection Performance of Metal Disc-PZT Attachment Method

**Figure 10:**
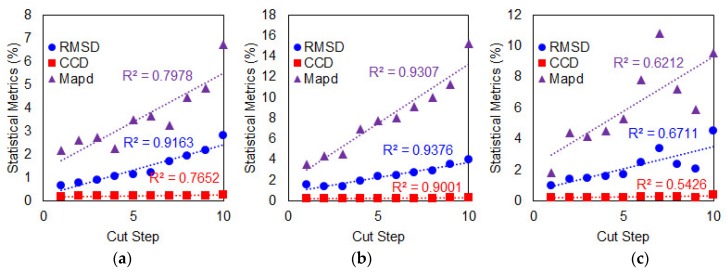
Performance evaluation of metal disc PZT attachment method subject to damage (**a**) Case 1; (**b**) Case 2; (**c**) Case 3

For a delamination defect or Case 1 as shown in Figure 10a, the RMSD shows the best performance among the three metrics with R^2^ = 0.9163 followed by MAPD with R^2^ = 0.7978 and then CCD with R^2^ = 0.7652. For Case 2 as shown in Figure 10b, the RMSD has the best performance in detecting increasing crack damage at fixed location with R^2^ = 0.9376 compared to MAPD and CCD with R^2^ = 0.9307 and 0.9001, respectively. Finally, for Case 3 as shown in Figure 10c, again the RMSD with R^2^ = 0.6711 performed the best in detecting progressive crack damage towards the PZT patch followed by MAPD and CCD with R^2^ = 0.6212 and 0.5426, respectively. Based on the three cases above, only the results for Case 2 can be considered to be excellent since all three metrics have R^2^ coefficients above 0.9. In fact, performance of the metal disc-PZT is the best among the three attachment methods considered in this study when used to detect increasing crack damage at a fixed location using all three metrics. Including the results from Figure 8 and Figure 9, although that the RMSD values generally show best correlation with increase in all damage types, the MAPD values have significantly higher values. This shows that an effective monitoring approach would be to use both the RMSD and MAPD metrics as MAPD would be best used to identify if damage has occurred where RMSD could be used to detect the location of damage.

## 6. Finite Element Modelling and Simulation

**Figure 11:**
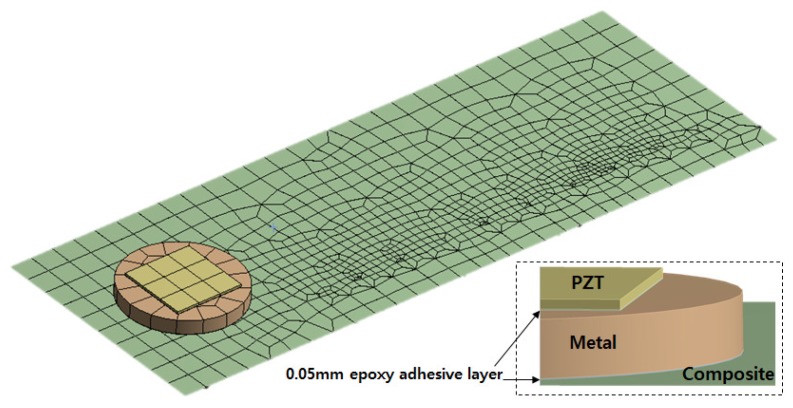
FEA model of the test specimen (metal disc PZT attachment method).

**Figure 12:**
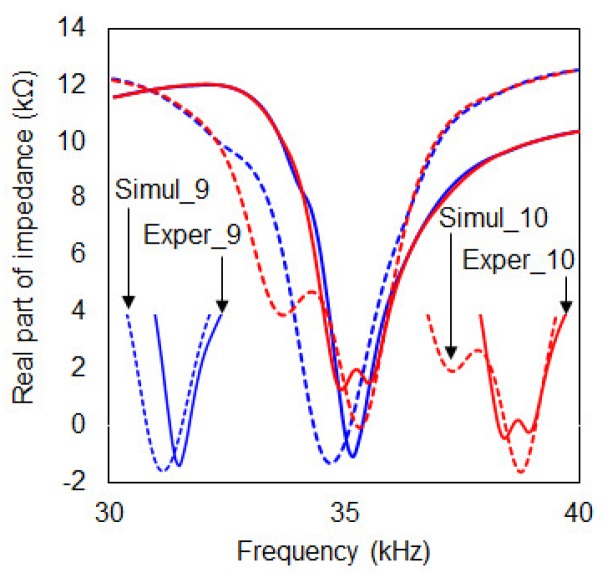
FEA simulation results versus experimental results (cut steps 9 and 10).

For verification of the experimental results, a FEA tool, the ANSYS software, was used for the remaining part of this study. Coupled field analysis (CFA) was performed to simulate the metal disc-PZT attachment method conducted in this study where all the mechanical properties of the components for the simulation can be found in Table 4. The FEA model in Figure 11 was created with 7274 nodes and 967 elements where the same damage identical to damage case 3 was simulated. Thus the model has 10 cuts of 10 mm length causing irregularity of the meshes as shown in the figure. Since a large resonance was observed between 30 kHz and 40 kHz, this frequency range was used for the simulation. Using the FEA model, the two simulation cases conducted were with cut steps 9 and 10 to the composite plate where the result can be seen in Figure 12 in the frequency range from 30 kHz to 40 kHz. Looking at the bottom left part of the figure, it can be noticed for the cut step 9 case that the simulation result (“Simul_9”) has a resonance peak lower than the experimental result (“Exper_9”). For the cut step 10 case, the extra damage has caused an additional resonance peak resulting in two different peaks. The difference between the two impedance signatures “Simul_10” and “Exper_10” is the resonance frequency range. The experiment has caused two small peaks close together around 39 kHz where the simulation result shows one peak at around 36 kHz and the other at 39 kHz.

## 7. Conclusions

In this study, the detection capabilities and performance of the EMI technique have been investigated. Glass fibre composite plates with various PZT attachment methods have been prepared and detection of common defects such as delamination and cracks with the EMI technique under study has been performed. The conventional attachment for PZT was made by directly attaching it to a composite plate. In order to ensure multiple peaks were generated throughout the frequency range of impedance signature when performing the EMI measurement, a thinner composite plate was used in this study. The second PZT attachment method was using a steel wire as a medium between PZT and the plate, which can be conducted on any type of surfaces. The last method under study was to overcome the problem of peakless impedance signatures by attaching PZT to a metal disc that was a part of the plate.

- From the results, it was confirmed that the use of a thinner plate influences the performance of the conventional attachment method due to existence of multiple peaks in the frequency range of the impedance signatures. The conventional PZT attachment method was reliable for detecting a delamination defect (Case 1) and progressive crack damage (Case 3) when quantified using the three different statistical metrics, namely RMSD, MAPD and CCD.
- Overall, the performance of the steel wire-PZT attachment method was quite good in all three cases of damage types, especially when the damage was quantified using RMSD and MAPD. However, no relationship was found between CCD values and any of the three damage types. The steel wire-PZT attachment method performed better than the conventional PZT attachment method using RMSD and MAPD metrics in detecting increasing crack damage at a fixed location (Case 2).
- Finally, one can conclude that an effective way of monitoring a thin composite structure would be to use both the RMSD and MAPD values as the results showed that the RMSD has best R2 values where MAPD values better indicated the existence of damage.

## Figures and Tables

**Table 1:** RMSD, MAPD and CCD values (%) calculated for conventional PZT attachment method.

Cut	Case 1			Case 2			Case 3		
Step	RMSD	MAPD	CCD	RMSD	MAPD	CCD	RMSD	MAPD	CCD
1	1.53	5.13	0.21	3.31	0.26	13.20	2.09	0.22	7.87
2	1.63	4.80	0.21	4.77	0.32	36.27	3.36	0.26	15.47
3	2.50	7.03	0.24	4.85	0.33	37.18	4.08	0.29	19.64
4	3.87	13.18	0.28	4.77	0.32	42.35	4.34	0.30	23.38
5	3.21	17.13	0.26	4.97	0.33	52.92	4.17	0.29	25.50
6	5.26	19.22	0.36	4.55	0.31	40.36	4.77	0.32	26.68
7	6.02	22.96	0.40	5.26	0.35	25.85	4.93	0.33	24.25
8	6.43	30.24	0.43	4.93	0.33	28.66	5.52	0.37	27.62
9	6.06	18.90	0.40	5.31	0.35	30.50	4.92	0.33	27.06
10	6.64	27.85	0.43	5.45	0.36	31.59	5.12	0.34	29.18

**Table 2:** RMSD, MAPD and CCD values (%) calculated for steel wire PZT attachment method.

Cut	Case 1			Case 2			Case 3		
Step	RMSD	MAPD	CCD	RMSD	MAPD	CCD	RMSD	MAPD	CCD
1	0.24	0.20	0.77	0.22	0.20	1.72	0.23	0.20	0.61
2	0.25	0.20	0.67	0.25	0.20	1.32	0.24	0.20	0.68
3	0.25	0.20	0.56	0.28	0.20	1.46	0.32	0.20	1.20
4	0.32	0.20	0.93	0.33	0.20	1.42	0.40	0.20	1.37
5	0.35	0.20	0.86	0.35	0.20	2.64	0.38	0.20	1.27
6	0.44	0.20	1.31	0.36	0.20	2.97	0.41	0.20	1.22
7	0.42	0.20	0.81	0.39	0.20	2.87	0.46	0.20	1.47
8	0.44	0.20	1.71	0.40	0.20	2.86	0.43	0.20	2.20
9	0.43	0.20	1.37	0.42	0.20	3.13	0.42	0.20	2.33
10	0.47	0.20	1.79	0.47	0.20	3.65	0.40	0.20	2.38

**Table 3:** RMSD, MAPD and CCD values (%) calculated for metal disc PZT attachment method.

Cut	Case 1			Case 2			Case 3		
Step	RMSD	MAPD	CCD	RMSD	MAPD	CCD	RMSD	MAPD	CCD
1	0.68	0.20	2.16	1.57	0.22	3.59	0.97	0.21	1.86
2	0.78	0.21	2.61	1.38	0.21	4.35	1.41	0.22	4.39
3	0.92	0.21	2.72	1.39	0.21	4.53	1.48	0.22	4.16
4	1.08	0.21	2.26	1.92	0.23	7.01	1.59	0.22	4.55
5	1.14	0.21	3.50	2.43	0.24	7.75	1.71	0.23	5.29
6	1.24	0.21	3.64	2.48	0.25	8.06	2.47	0.25	7.83
7	1.69	0.23	3.25	2.76	0.26	9.12	3.38	0.30	10.8
8	1.95	0.23	4.44	2.96	0.26	10.03	2.38	0.25	7.19
9	2.17	0.24	4.84	3.54	0.29	11.26	2.10	0.24	5.89
10	2.82	0.27	6.73	3.98	0.31	15.27	4.51	0.38	9.58

**Table 4:** Properties of the PZT for ANSYS simulation.

PSI-5A4E		
**Density**	7800	
**Damping Ratio**	0.0125	
**Stiffness Matrix [*c^E^*]**	*C*_11_ = *C*_22_	152
	*C*_12_	102
	*C*_13_ = *C*_23_	100
	*C*_33_	127
	*C*_44_ = *C*_55_	21
	*C_66_*	25
**Piezoelectric Stress Matrix [*e*]**	*e*_31_ = *e*_32_	–5.5
	*e_33_*	16.4
	*e*_24_ = *e*_15_	12.4
**Electric Permittivity Matrix [*ε^s^*]**	*ε_11_* = *ε_22_*	950
	*ε_33_*	890
